# Development of a HPLC Method for the Quantitative Determination of Capsaicin in Collagen Sponge

**DOI:** 10.1155/2015/912631

**Published:** 2015-11-03

**Authors:** Chun-Lian Guo, Hong-Ying Chen, Bi-Ling Cui, Yu-Huan Chen, Yan-Fang Zhou, Xin-Sheng Peng, Qin Wang

**Affiliations:** School of Pharmacy, Guangdong Medical University, Dongguan, Guangdong 523808, China

## Abstract

Controlling the concentration of drugs in pharmaceutical products is essential to patient's safety. In this study, a simple and sensitive HPLC method is developed to quantitatively analyze capsaicin in collagen sponge. The capsaicin from sponge was extracted for 30 min with ultrasonic wave extraction technique and methanol was used as solvent. The chromatographic method was performed by using isocratic system composed of acetonitrile-water (70 : 30) with a flow rate of 1 mL/min and the detection wavelength was at 280 nm. Capsaicin can be successfully separated with good linearity (the regression equation is *A* = 9.7182*C* + 0.8547; *R* ^2^ = 1.0) and perfect recovery (99.72%). The mean capsaicin concentration in collagen sponge was 49.32 mg/g (RSD = 1.30%; *n* = 3). In conclusion, the ultrasonic wave extraction method is simple and the extracting efficiency is high. The HPLC assay has excellent sensitivity and specificity and is a convenient method for capsaicin detection in collagen sponge. This paper firstly discusses the quantitative analysis of capsaicin in collagen sponge.

## 1. Introduction

**Figure 1:**
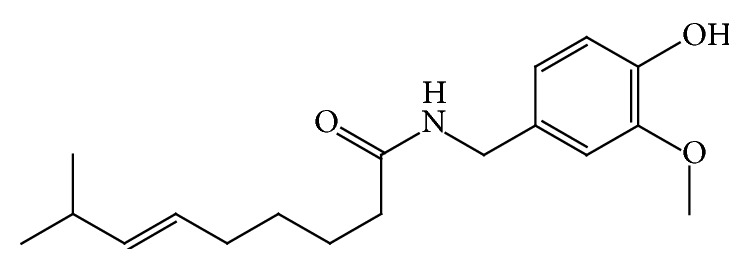
Molecular structure of capsaicin.

Capsaicin (*trans*-8-methy-*N*-vanilly-6-nonenamide in Figure 1) is a valuable pharmacological agent with effective pain management for rheumatoid arthritis, osteoarthritis, neuralgias, and diabetic neuropathy [1]. Yet capsaicin is actually irritant to human skin producing a burning sensation [2], and because of its high degree of first-pass metabolism in gastric intestinal track and short half-life by intravenous administration (7.06 min) [3, 4], transdermal delivery system seems to be more advantageous for capsaicin. In our previous research, we chose cataplasm [5], cubic phase gel [6, 7], and cubosome [8, 9] as transdermal delivery system providing the sustained release for capsaicin.

Recent studies and case reports in the literature have indicated that capsaicin can directly inhibit the proliferation of fibroblasts and collagen synthesis, showing special preventing and curing effects on hypertrophic scar [10]. Capsaicin also takes the role of antiscar hyperplasia by exhausting neuropeptides SP [11]. We are interested in the inhibition effect on the proliferation of fibroblasts and collagen synthesis and try to choose the collagen as novel scaffolds to the wound healing for capsaicin.

Collagen is a natural polymer of particular interest for drug delivery system since collagen is a major natural constituent of connective tissue and a major structural protein of any organs [12]. Biomaterials made of collagen offer several advantages: they are biocompatible, nontoxic, and fully biodegradable [13], which eliminates the need of secondary surgery to remove the carrier [14]. Zhao et al. [15] reported that DNA-collagen carrier can control the slow release of silver ions* in vivo*. Ruszczak and Friess [14] demonstrated that the drug release process ensures relatively stable serum concentrations, prolonging drug efficacy and increasing significantly the rate of wound healing [14] when collagen was used as a carrier for topical antibiotic therapy.

LC-MS/MS and HPLC methodology were reported for the determination of capsaicin in different applications such as liposomes, pellets [16–18]. As far as we know, there were no reports about the collagen sponge capsaicin loaded. Our previous experiment has proved that scaffold could decrease the irritation of capsaicin to the wound and inhibit the formation of hypertrophic scar. Based on the experiment, a patent application was filed in China (CN104490760A). Here we developed the HPLC method to determine the capsaicin concentration in collagen sponge.

## 2. Experimental

### 2.1. Reagents and Chemicals

Fresh bovine tendon was collected from Yuanling Supermarket (Dongguan, China, batch number 20140411). Pepsin was purchased from Sigma. Capsaicin was purchased from Wuhan Hengshuo Technology Development Co. Ltd. (Wuhan, Hubei, China). Capsaicin standard was purchased from the institution for the control of pharmaceutical and biological products of China (batch number 110839-201205). Acetonitrile (high performance liquid chromatographic grade) was purchased from Tianjin Kermel Chemical Reagent Co., Ltd. (Tianjin, China, batch number 20120210). Water purified through flow water purification system (Qingdao, China) was used throughout this study. All other reagents were of HPLC or analytical grade and used as received.

### 2.2. Apparatus

Analyses were performed on UV-6000s Spectrophotometer (Shanghai Metash Instruments Co., Ltd.) and LC-20A High Performance Liquid Chromatograph with SPD-20A UV-detector (Shimadzu Instruments Co., Ltd., Japan).

### 2.3. Preparation of Capsaicin-Collagen Sponge

Fresh bovine tendon was firstly cleaned and washed twice in 10% NaCl solutions to remove residual proteins on the surface. Then the bovine tendon was washed three times with distilled water and treated with 0.5 M acetic acid solution (containing 0.08% (w/v) pepsin) for a period of 72 h under magnetic stirring in cold room (4°C); the insoluble part of bovine tendon was filtered out. 0.9 M NaCl was used to salt out the collagen from the supernatant by keeping it undisturbed for 24 h at 4°C. Next day the suspension was centrifuged at 8000 rpm for 5 min at 4°C and the precipitate was resolubilized in 0.5 M acetic acid. The final content was then dialyzed against 0.1 M acetic acid and distilled water, respectively, for 24 h. Before the process of freeze-drying, capsaicin was dissolved in ethanol solution; the capsaicin ethanol solution and glycerin were, respectively, added in the collagen solution and vortex-mixed to achieve a homogeneous state. Then the samples were later freeze-dried and capsaicin-collage sponge was obtained. The final concentration of capsaicin and glycerin was 0.05% and 5% w/w, respectively.

### 2.4. Chromatographic Conditions

The analytical column was Inertsustain C_18_ column (150 mm × 4.6 mm, 5 *μ*m). The mobile phase was acetonitrile-water (70 : 30) with a flow rate of 1 mL/min, and the detection wavelength was at 280 nm. The sample injection volume was 20 *μ*L. The column temperature was maintained at 40°C.

### 2.5. Preparation of Standard Solutions

6.16 mg capsaicin was transferred into 10 mL volumetric flask, dissolved, and made up to volume with acetonitrile. This solution was further diluted with acetonitrile to yield solutions containing 616, 308, 154, 77, 38.5, and 19.25 *μ*g/mL.

### 2.6. Sample Preparation Procedure

Approximately 50 mg capsaicin-collagen sponge was weighed carefully to the vials; then the sample was ultrasonically extracted with methanol (20 mL) as extraction solvent for 30 min. After that, the extraction solutions were filtered using 0.22 *μ*m filter membrane and 20 *μ*L aliquot was injected into the HPLC system.

## 3. Results and Discussion

### 3.1. Preparation of Capsaicin-Collagen Sponge

**Figure 2:**
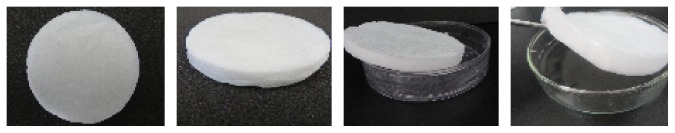
Photograph of capsaicin-collagen sponge.

White and soft collagen sponges were obtained (Figure 2). Many little porous and dense structures were observed from the top-surface and in the case of cross section of the scaffolds under scanning electron microscope (data not shown).

### 3.2. Chromatographic Conditions

**Figure 3:**
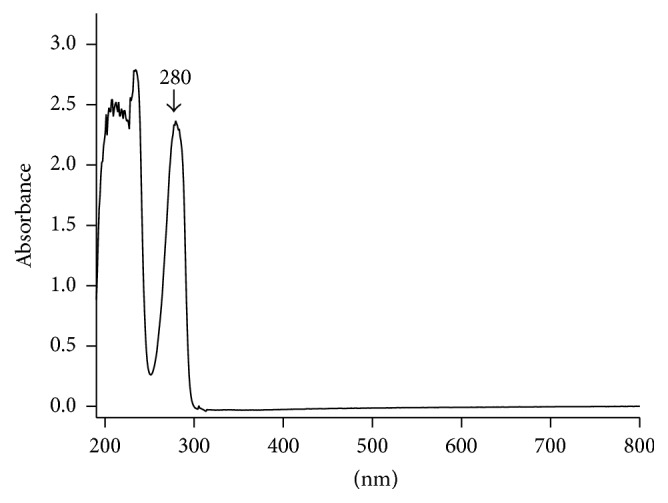
UV spectrum of capsaicin.

**Figure 4:**
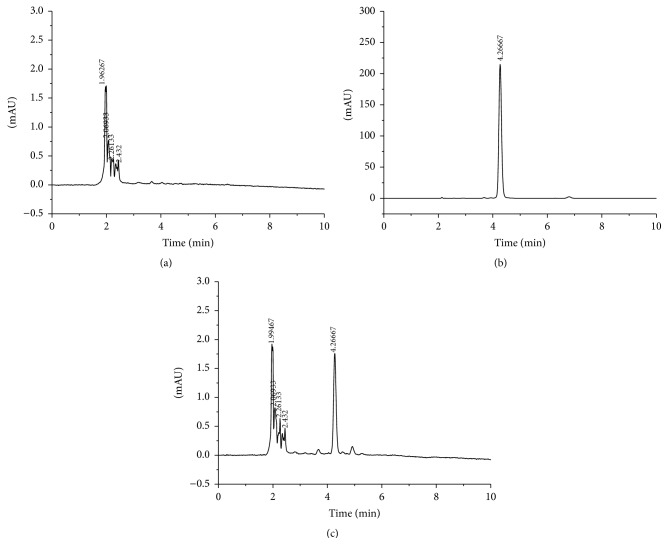
HPLC chromatogram of blank collagen sponge (a), standard capsaicin (b), and capsaicin-collagen sponge (c).

UV spectrum of capsaicin showed maximum absorbance at 280 nm wavelength (Figure 3). Therefore, 280 nm was selected as the detection wavelength. In selected spectrometry conditions, chromatography symmetry of capsaicin was with higher degrees (Figure 4). The response was high and the retention time was less than 6 min with good reproducibility.

### 3.3. Specificity

Blank collagen sponge sample was prepared according to Section 2.3 (no capsaicin), and the chromatogram was shown in Figure 4(a). A certain concentration of capsaicin standard solution as well as capsaicin-collagen sponge sample solution was injected into the HPLC system with the same operation, and the chromatogram was shown in Figures 4(b) and 4(c). The retention time was 4.25 min. Obviously, the peaks of the capsaicin were well separated and were not affected by the excipients.

### 3.4. Linearity

**Figure 5:**
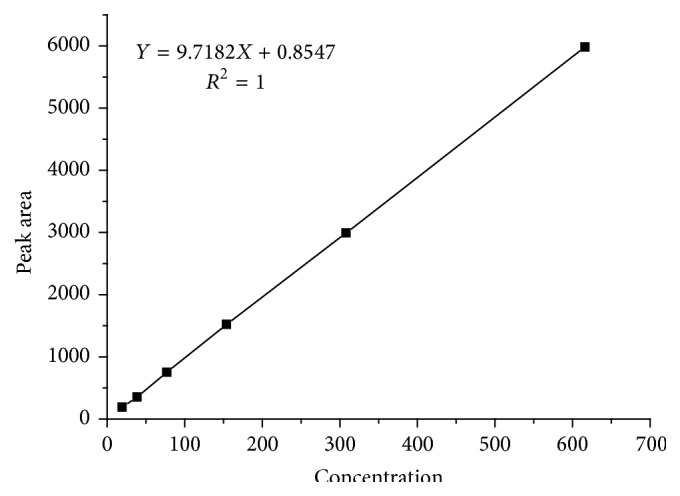
Capsaicin calibration curve.

The calibration curves for capsaicin were found to be linear within the range of 19.25 to 616.0 *μ*g/mL (shown in Figure 5). The regression equation was *A* = 9.7182*C* + 0.8547 (*R* ^2^ = 1.0), where *A* is peak area and *C* is the concentration (*μ*g/mL) of capsaicin standard solution. The correlation coefficient indicated a good linear relationship between peak area and concentration over a wide range.

### 3.5. Precision

Precision was demonstrated at 3 concentration levels in intraday and interday studies. Intraday precision was determined by injection of capsaicin standard solutions on the same day. Interday precision was checked by repeating the studies on two different days. The intraday and the interday precisions of capsaicin were found to be acceptable (RSD < 2%).

### 3.6. Recovery

Mean recovery for capsaicin at different concentration levels was found to be 97.69 ± 1.54% (RSD = 1.57%; *n* = 3), 100.45 ± 1.30% (RSD = 1.3%; *n* = 3), and 101.04 ± 0.48% (RSD = 0.47%; *n* = 3), respectively. The whole average recovery rate is 99.72 ± 1.87% (RSD = 1.87%; *n* = 9). Recovery test data is shown in Table 1. The low values of RSD revealed that the present method was accurate, reliable, and reproducible.

### 3.7. The Stability Experiment

The peak area of one sample was detected at different time intervals: 0, 2, 6, 12, 24, 48, and 72 h, with the following results being obtained: 5106.93, 5095.17, 5093.92, 5111.11, 5118.78, 5111.93, and 5331.92. The mean peak area was 5138.54, RSD = 1.67%, and the data obtained show samples were stable within 72 h.

### 3.8. Limits of Detection (LOD) and Quantification (LOQ)

The LOD (*S*/*N* = 3) and LOQ (*S*/*N* = 10) were determined at 2.09 *μ*g/mL and 6.74 *μ*g/mL, respectively.

### 3.9. Content of Capsaicin in Collagen Sponge

Capsaicin was determined with the proposed method in collagen sponge. The mean concentration was 49.32 mg/g (RSD = 1.30%; *n* = 3).

## 4. Discussion

**Figure 6:**
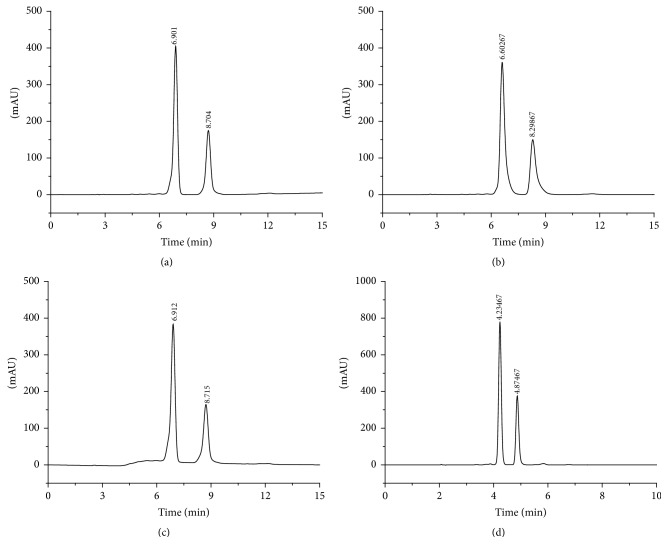
HPLC chromatogram of capsaicin in different mobile phase condition. (a) Methanol : water = 70 : 30, (b) methanol : water : H_3_PO_4_ = 70 : 30 : 0.1, (c) methanol : water : triethylamine = 70 : 30 : 0.05, and (d) acetonitrile : water = 70 : 30.

Several mobile phase systems such as methanol-water and methanol-water-H_3_PO_4_ systems and methanol-water-triethylamine have been tested in this study. However, the chromatography symmetry of capsaicin is not so good compared to acetonitrile-water system (Figure 6).

The chromatographic method was eventually carried out using an isocratic system with a mobile phase of acetonitrile-water (70 : 30) applied at a flow rate of 1 mL/min with detection wavelength at 280 nm. Under the mobile phase conditions, elution of analyte was completed in less than 6 min and retention time of capsaicin was 4.25 min. The method was validated with the parameters of specificity, linearity, sensitivity, accuracy, precision, and reproducibility. All data shows that the method is accurate within the desired ranges.

**Figure 7:**
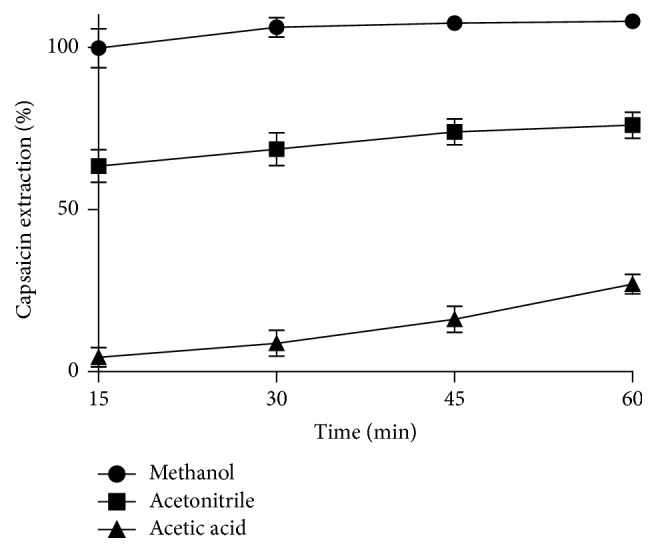
The effect of different solvents and extraction time on capsaicin extraction.

Considering the characteristic of capsaicin as a hydrophobic drug with weak alkaline, the influence of different solvents (acetic acid, acetonitrile, and methanol) and extracting time (15, 30, 45, and 60 min) on capsaicin extraction efficiency was investigated. Shown in Figure 7, the extracting efficiency was positively influenced by the extracting time: as ultrasonication time increases, the capsaicin extraction increases. The capsaicin extraction rate reaches 100% after 30 min of ultrasonication in methanol. However the extraction efficiency is much lower for the case when acetonitrile (76.82%, 60 min) and acetic acid (25.92%, 60 min) were chosen as the solvent. Collagen sponge and capsaicin could be dissolved by acetate, but they could precipitate in the acetonitrile-water system. That is why the free capsaicin is so low as the acetic acid was used as the extraction solvent. Therefore, methanol was the optimum solvent and ultrasonic time is 30 min.

## 5. Conclusion

In this work, we used HPLC method to separate and determine the capsaicin in collagen sponge. Ultrasonic wave extraction method was used to extract capsaicin from collagen sponge and methanol was used as the solvent. The ultrasonic wave extraction method is simple; the extracting efficiency of capsaicin from sponge reaches 100% after ultrasonication for 30 min. The linear range of capsaicin concentration was 19.25 to 616.0 *μ*g/mL. Calculated by samples, the intra- and interassay precision (RSD) were less than 2%. The average recovery rate was 99.72%. Capsaicin peaks were well resolved and free from tailing (<1.5). This method was sensitive with good precision and accuracy. The separation time was 4.25 min and the excipients did not interfere with the detection of capsaicin. Capsaicin amount determined was 49.32 mg/g (RSD = 1.30%; *n* = 3) with the above method in collagen sponge. The present study is the first report on the capsaicin determination combined with collagen sponge system. The method can be used for controlling the quality of the capsaicin-collagen sponge and can be helpful for further investigation.

## Figures and Tables

**Table 1:** Recovery test (*n* = 3).

Added (mg)	Found (mg)	Recovery (%)	Mean ± SD	RSD (%)
	0.0188	97.90		
0.019	0.0185	96.05	97.68 ± 1.54	1.57
	0.0191	99.10		
	0.1525	99.03		
0.154	0.1551	100.74	100.45 ± 1.30	1.29
	0.1564	101.59		
	0.6196	100.58		
0.616	0.6222	101.01	101.04 ± 0.48	0.47
	0.6254	101.53		
